# Aortic Pressure Levels and Waveform Indexes in People Living With Human Immunodeficiency Virus: Impact of Calibration Method on the Differences With Respect to Non-HIV Subjects and Optimal Values

**DOI:** 10.3389/fcvm.2021.772912

**Published:** 2021-12-23

**Authors:** Alejandro Diaz, Marina Grand, Juan Torrado, Federico Salazar, Yanina Zócalo, Daniel Bia

**Affiliations:** ^1^Consejo Nacional de Investigaciones Científicas y Técnicas, Instituto de Investigación en Ciencias de la Salud (IICS), Universidad Nacional del Centro de la Provincia de Buenos Aires (UNICEN), Tandil, Argentina; ^2^Instituto de Investigación en Ciencias de la Salud, Facultad de Ciencias de la Salud, Universidad Nacional del Centro de la Provincia de Buenos Aires, Tandil, Argentina; ^3^Hospital Dr. Héctor M. Cura, Olavarría, Argentina; ^4^Department of Internal Medicine, Jacobi Medical Center, Albert Einstein College of Medicine, New York, NY, United States; ^5^Servicio de Cardiología, Hospital Privado de Comunidad, Mar del Plata, Argentina; ^6^Departamento de Fisiología, Facultad de Medicina, Centro Universitario de Investigación, Innovación y Diagnóstico Arterial, Universidad de la República, Montevideo, Uruguay

**Keywords:** aortic pressure, calibration, human immunodeficiency virus, pulse wave analysis, wave separation analysis, pulse contour analysis

## Abstract

**Background:** There are scarce and controversial data on whether human immunodeficiency virus (HIV) infection is associated with changes in aortic pressure (aoBP) and waveform-derived indexes. Moreover, it remains unknown whether potential differences in aoBP and waveform indexes between people living with HIV (PLWHIV) and subjects without HIV (HIV-) would be affected by the calibration method of the pressure waveform.

**Aims:** To determine: (i) whether PLWHIV present differences in aoBP and waveform-derived indexes compared to HIV- subjects; (ii) the relative impact of both HIV infection and cardiovascular risk factors (CRFs) on aoBP and waveform-derived indexes; (iii) whether the results of the first and second aims are affected by the calibration method.

**Methods:** Three groups were included: (i) PLWHIV (n = 86), (ii) HIV- subjects (general population; *n* = 1,000) and (iii) a Reference Group (healthy, non-exposed to CRFs; *n* = 398). Haemodynamic parameters, brachial pressure (baBP; systolic: baSBP; diastolic: baDBP; mean oscillometric: baMBPosc) and aoBP and waveform-derived indexes were obtained. Brachial mean calculated (baMBPcalc=baDBP+[baSBP-baDBP]/3) pressure was quantified. Three waveform calibration schemes were used: systolic-diastolic, calculated (baMBPcalc/baDBP) and oscillometric mean (baMBPosc/baDBP).

**Results:** Regardless of CRFs and baBP, PLWHIV presented a tendency of having lower aoBP and waveform-derived indexes which clearly reached statistical significance when using the baMBPosc/baDBP or baMBPcalc/baDBP calibration. HIV status exceeded the relative weight of other CRFs as explanatory variables, being the main explanatory variable for variations in central hemodynamics when using the baMBPosc/baDBP, followed by the baMBPcalc/baDBP calibration.

**Conclusions:** The peripheral waveform calibration approach is an important determinant to reveal differences in central hemodynamics in PLWHIV.

## Introduction

Global mortality in people living with human immunodeficiency virus (PLWHIV or HIV+) has dramatically decreased over the last years due to significant improvements in both the access to highly active antiretroviral therapy and clinical care (1). However, these achievements were challenged by the higher risk of cardiovascular disease that experience these patients compared to non-HIV subjects (HIV-) (2, 3). HIV-related cardiovascular disease risk is thought to be multifactorial, involving the development of a pro-inflammatory state associated with the chronic infection (4), the use of highly active antiretroviral therapy with an adverse metabolic profile (5), and the HIV-related high prevalence of other cardiovascular risk factors (CRFs) (6). Accordingly, PLWHIV on highly active antiretroviral therapy have shown an elevated prevalence of other CRFs, as well as premature cardiovascular disease reflected by a high prevalence of early arterial alterations (e.g., increased arterial stiffness, impaired vascular reactivity) (7, 8). However, the impact of HIV infection itself on some cardiovascular variables remains controversial. More precisely, there are scarce, and above all, controversial data on whether HIV infection is independently associated with aortic blood pressure (aoBP) levels and waveform-derived indexes. Previous studies have reported that both aoBP and some waveform-derived indexes were either elevated (9, 10), unchanged (9, 11), or even reduced (12–14) in PLWHIV compared to HIV- subjects.

At least three factors could explain these controversies. First, prior studies that have reported aoBP levels and waveform-derived indexes in PLWHIV compared to HIV- subjects either (i) did not adjust for any CRF (9, 15) or (ii) only adjusted for some variables (e.g., age, sex, body mass index) without considering other cofactors such as cholesterol, triglycerides and diabetes (10–14). Thus, it remains to be determined at what extent potential differences in central hemodynamics associated with HIV infection would be directly related to the disease condition and/or would be determined by the presence of concomitant CRFs. Second, certain studies have considered control groups (HIV-) with relatively small sample size (e.g., *n* = 26–37) (9, 10, 15), which significantly reduces the statistical power of the tests to find significant differences between the groups (Type-II error). Last, these studies have not considered the relevance of the calibration of the pressure waveform at the time of assessing non-invasively central hemodynamics (16). Several studies that have compared the aoBP between PLWHIV and HIV- subjects either did not report the calibration method (9, 10, 13, 15) or used currently not recommended schemes (i.e., systolic-diastolic calibration; see below) (11, 14).

Non-invasive estimation of aoBP can be achieved by several devices, which differ in multiple features such as the principle applied to assess the pressure or surrogate signals, the arterial site for pulse waveform recording and/or the model or mathematical analysis considered to obtain central hemodynamic data (16). Most of the devices use oscillometry/plethysmography, applanation tonometry, or ultrasonography to obtain pulse waveforms from radial, brachial or carotid arteries. Then, from the acquired pulse waveform and posterior calibration, the devices quantify aoBP directly (i.e., calibration of carotid waveforms) or indirectly (e.g., applying generalized transfer functions to brachial or radial waveforms) (16–29). In both cases, research and clinical practice have mostly used two different brachial artery blood pressure (baBP)-derived calibration schemes, (i) calibration to brachial systolic (baSBP) and diastolic (baDBP) pressure (“systolic-diastolic calibration”) and calibration to baDBP and brachial mean blood pressure (baMBP) (“baMBP/baDBP calibration”) (27–29). It is noteworthy, that baMBP levels to be used for calibration could be measured directly by oscillometry (baMBPosc; “baMBPosc/baDBP calibration”) or calculated (baMBPcalc; “baMBPcalc/baDBP calibration”) from baSBP and baDBP, using different scaling forms (e.g., classically a form factor equal to 0.33) (27–29). Previous studies have strongly recommended to describe in detail the calibration scheme used during the hemodynamic measures, since it is essential when interpreting the results at the time of evaluating for statistical differences and when assessing the potential clinical value of quantifying central over peripheral parameters (16, 19, 21, 22, 25, 27, 28). In previous studies performed on subjects of the general population (children, adolescents and adults) we showed that calibration with the “baMBPosc/baDBP” scheme resulted in higher aoBP values and stronger association between aoBP and cardiac structural properties (27–29). In this context, it remains unknown whether potential differences in aoBP levels and waveform-derived indexes between PLWHIV and HIV- subjects would be significantly affected by the calibration method. At least in theory, the calibration scheme could mask the existence of differences between of central hemodynamic indexes measured in PLWHIV vs. HIV- subjects.

This study sought to determine: (1) whether PLWHIV present differences in aoBP levels and waveform-derived indexes, compared to HIV- subjects, matched by demographic, anthropometric and levels of exposure to CRFs; (2) the impact to which HIV infection itself and its treatment vs. classical CRFs contribute to the levels of affectation (deviation from the age-related expected [optimal] value) for the different aoBP levels and waveform-derived indexes; (3) to what extent the results of the first and second aims are affected by the calibration method.

## Materials and Methods

### Study Population

This study was carried out in the context of the “Tandil Cardiovascular Project” (30–37), a population-based study developed in Provincia de Buenos Aires, Argentina. From this database, three different groups were assessed: (i) PLWHIV (*n* = 86), (ii) age range-matched non-HIV subjects (general population; *n* = 1,000) and (iii) a reference group (healthy subjects non-exposed to CRFs; *n* = 398). The reference group was selected to quantify differences between measured and “expected” values (see below). All procedures were conducted in agreement with the Declaration of Helsinki. The study protocol was approved by the Institution's Ethics Committee. Written informed consent was obtained prior to the evaluation.

### PLWHIV Group

In addition to the general variables, the following data were obtained from the PLWHIV' electronic medical record: time since HIV diagnosis, highly active antiretroviral therapy exposure and time under this therapy and/or protease inhibitors, history of opportunistic infection, CDC's HIV category, HIV viral load < 50 copies/ml (%), and CD4+ lymphocytes count (cells/mm^3^). Subjects under chronic treatment with steroids or chemotherapy, and pregnant women, were excluded.

### Clinical Evaluation

Blood samples were obtained after 9–12 h of fasting. Subject's body height and weight were measured and body mass index was calculated (weight/height^2^). Dyslipidemia, diabetes and hypertension were considered present if they had been previously diagnosed by referring physicians or the patient was receiving lipid-lowering, glucose-lowering or antihypertensive drugs (38). Diabetes was diagnosed based on abnormal plasma glucose levels (39). Dyslipidemia was defined as total cholesterol >240 mg/dL, low-density lipoprotein cholesterol >160 mg/dL, high-density lipoprotein cholesterol for men <40 mg/ and for women <50 mg/dL and/or triglycerides >250 mg/dL (40). Smokers (defined as usually smoking at least one cigarette/week) were identified. Obesity was defined as body mass index ≥30 kg/m^2^.

### Cardiovascular Evaluation

Participants were asked to avoid exercise, tobacco, alcohol, caffeine, and food-intake four hours before the evaluation. All hemodynamic measurements were performed in a temperature-controlled room (21–23°C), with the subject in supine position and after resting for at least 10–15 min.

Peripheral baBP levels [baSBP, baDBP and baMBPosc (lowest cuff pressure at which the oscillations are maximal)] and waveforms were obtained by a brachial cuff based oscillometric device (Mobil-O-Graph system, I.E.M, Stolberg, Germany) (20, 29). The brachial pulse pressure (baPP, baPP=baSBP-baDBP) and baMBPcalc (baMBPcalc=baDBP+[baPP]/3) were obtained. Once baBP is measured, the cuff is instantly inflated, and baBP waveforms are recorded for 10 s. Subsequently, the device determines the aoBP levels and waveforms from peripheral recordings.

By means of pulse wave analysis, wave separation analysis and pulse contour analysis, the following variables were obtained: (i) aortic systolic, diastolic and pulse pressure (aoSBP, aoDBP and aoPP); (ii) heart rate; (iii) maximal amplitude of forward-traveling (Pf) and backward-traveling (Pb) wave components, and Pb/Pf ratio (Reflection Magnitude); (iv) first inflection pressure and time (difference in pressure and time, from the beginning of aoBP systolic phase [“foot wave”] to the first systolic inflection point [shoulder] in the aoBP waveform); (v) systolic time (duration of waveform-derived ejection phase); (vi) time from the “foot wave” to the peak or maximal amplitude of Pf and Pb components (TmaxForward and TmaxBackward); (vii) time of arrival of the backward wave (TstartBackward); (viii) aortic pulse wave velocity (aoPWV; calculated from the reconstructed aoBP waveform); (ix) augmentation index (AIx) and heart rate-adjusted AIx (AIxHR75)l; (x) stroke volume, cardiac output and index, and systemic vascular resistance (41). Definitive values were the average of at least six measures obtained in a single visit. Only high-quality recordings and satisfactory waves (visual inspection) were considered (29).

All values were quantified by using the three different methods of calibration: baSBP/baDBP, baMBPcalc/baDBPand baMBPosc/baDBP (27–29).

### Statistical Analysis

#### Cardiovascular Differences Between PLWHIV and HIV- Subjects

**Figure 1:**
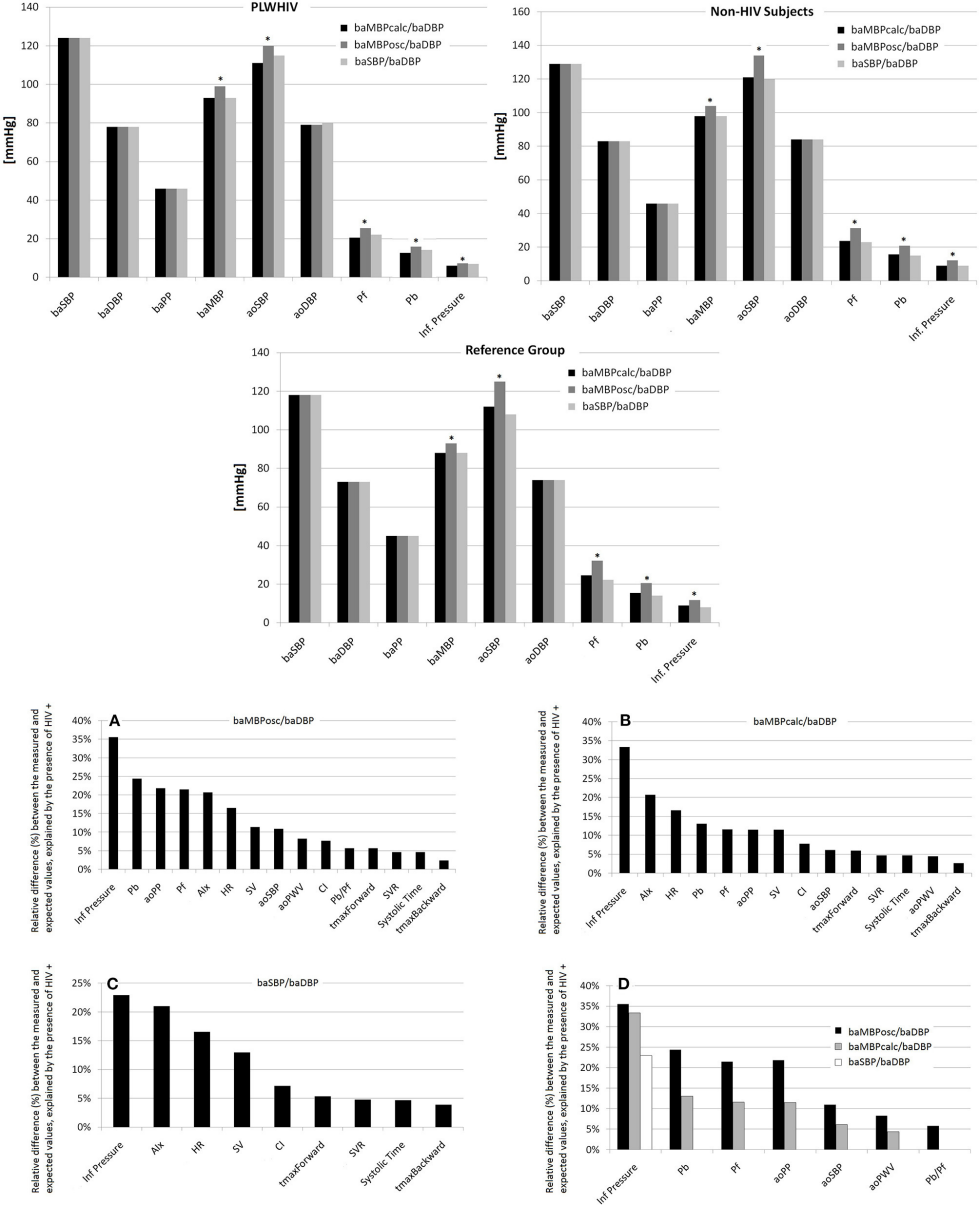
**(Top)** Comparison of hemodynamic levels obtained with different calibration schemes. *Represents *p* < 0.05 with respect to the same variable quantified using other calibration scheme. **(Bottom)** Percentage of variation between the measured and optimal (expected) value of the cardiovascular variables for each of the calibration schemes that could be explained by the HIV status. **(A–C)** Percentage of variation between the measured and expected (“optimal”) value of the cardiovascular variables for each of the calibration schemes that could be explained by the HIV status (values ranked from the highest to the lowest value). **(D)** Presents comparatively the differences depending on the calibration scheme used. Abbreviations in the text.

After analyzing the characteristics of the included groups (Table 1, Supplementary Table 1), we compared (in each subgroup) the central hemodynamic levels obtained with different calibration schemes (Figure 1; ANOVA+Bonferroni). After that, the cardiovascular properties of HIV+ and HIV- were compared (ANCOVA) adjusting for cofactors (demographic, anthropometric and CRFs) (Table 2, Supplementary Tables 2–4).

**Table 1:** Characteristics of included subjects: PLWHIV, Non-HIV group and Reference group.

	**PLWHIV**							**Non-HIV subjects (HIV -)**							**Reference group**						
**Variables**	**MV**	**SD**	**Min**	**p25**	**p50**	**p75**	**Max**	**MV**	**SD**	**Min**	**p25**	**p50**	**p75**	**Max**	**MV**	**SD**	**Min**	**p25**	**p50**	**p75**	**Max**
**Demographic, anthropometric, and clinical characteristics**																					
Female (%)				50.6				49.8				52.7									
Age (years)	44	12	19	36	44	53	75	51	14	19	41	52	62	75	38	13	19	28	38	46	74
Height (m)	1.65	0.10	1.45	1.58	1.65	1.72	1.89	1.68	0.10	1.42	1.61	1.68	1.75	2.00	1.69	0.10	1.42	1.61	1.69	1.75	2.00
Weight (kg)	72.3	17.1	42.6	57.0	71.6	82.0	119.2	79.9	17.6	37.0	68.0	79.0	90.0	159.0	67.9	12.5	37.0	59.0	67.0	75.0	113.0
BMI (kg/m^2^)	26.4	5.3	17.2	22.3	25.9	30.4	44.3	28.1	5.2	17.1	24.4	27.6	30.9	55.0	23.7	2.8	17.1	21.6	24.0	25.8	29.8
Htc (%)	40.9	4.9	25.6	38.0	40.9	44.4	53.0	42.0	3.3	26.0	40.0	42.0	44.0	55.0	41.7	2.3	36.0	40.0	42.0	43.0	46.0
Glyc (mg/dl)	101	29	67	90	96	103	298	101	29	55	87	97	107	427	81	10	65	72	78	88	109
Cr (mg/dl)	0.9	0.4	0.6	0.7	0.8	1.0	3.9	0.9	0.2	0.4	0.7	0.9	1.0	2.1	0.9	0.2	0.6	0.8	0.9	1.0	1.4
TC (mg/dl)	174	42	97	149	172	191	380	190	41	40	164	189	217	528	168	22	118	150	169	186	246
LDL (mg/dl)	104	26	45	89	100	123	160	114	34	24	89	116	136	248	106	26	48	86	108	124	166
HDL (mg/dl)	44	14	24	33	39	52	93	54	15	18	43	52	62	160	58	12	40	51	57	66	93
TG (mg/dl)	163	134	42	90	134	185	980	137	106	40	87	111	157	1,737	90	29	40	70	98	111	212
Obesity (%)				27.7				30.3				0.0									
HTN (%)				20.5				56.0				0.0			
Diabetes (%)				8.4				12.2				0.0									
Smoking (%)				38.6				12.8				0.0									
Dyslipidemia				13.3				51.7				0.0									
CVD (%)				2.4				3.4				0.0									
Antihypertensive (%)				14.5				47.6				0.0									
Anti-HLD drug (%)				13.3				28.0				0.0									
Antidiabetic drug (%)				7.3				9.0				0.0									
**PLWHIV-Related Clinical Characteristics**																					
Time HIV (mo)	50	71	0	6	19	64	303				————				————						
CDC:A1, A2, A3				24.7, 16.5, 10.6					————					————							
CDC: B1, B2, B3				3.5, 9.4, 5.9				————				————									
CDC: C3				29.4				————				————									
Hepatitis B and C				5.0 and 0.0				————				————									
HAART use				87.0				————				————									
NNRTIs				37.0				————				————									
NRTIs				33.0				————				————									
Abacavir				3.0				————				————									
PIs				1.0				————				————									
INSTIs				2.0				————				————									
HAART use (mo)	37	61	0	3	11	40	303				————				————						
Use of PIs (mo)	14	43	0	0	0	0	270				————				————						
PO infection				30.6				————				————									
HIV Viral Load				67.1				————				————									
CD4 count/ mm^3^	549	414	19	282	466	769	2,273				————				————						
IgG CMV +				58.8				————				————									

*MV, mean value; SD, standard deviation; Min, minimal; Max, maximal; p25, p50, p75, percentile 25, 50 and 75; Mo, months; BMI, body mass index; Hct, hematocrit; TC, LDL, HDL, total, low-density, and high-density lipoprotein cholesterol; TG, triglycerides; HTN, hypertension; CVD, cardiovascular disease; PO infection, previous opportunistic infection; PLWHIV, people living with HIV; Time HIV, time since HIV diagnosis; HAART, highly active antiretroviral therapy; NRTIs, nucleoside/nucleotide reverse transcriptase inhibitors; PIs, protease inhibitors; INSTIs, Integrase strand transfer inhibitors; CMV, cytomegalovirus; Cr, creatinine; Glyc, glycemia; A1, A2, A3, B1, B2, B3 and C3 refers to CDC HIV clinical categories*.

**Table 2:** Adjusted pairwise comparisons between PLWHIV and HIV- subjects (ANCOVA).

**Analysis of variables that do not depend on the calibration scheme**													
**Variables**	**Groups**		**MV**	**SE**	**Mean** **Difference**	***p*** **-value**	**Variables**	**Groups**		**MV**	**SE**	**Mean** **Difference**	***P*** **-value**
**Brachial pressure, heart rate and hemodynamic parameters**								**Aortic wave analysis-derived parameters**					
baSBP (mmHg)	Non-HIV		129.15	0.56	−0.75	0.710	AIx (%)	Non-HIV		23.32	0.54	−3.08	0.117
	PLWHIV		128.40	1.89				PLWHIV		20.24	1.84		
baDBP (mmHg)	Non-HIV		83.38	0.42	−1.94	0.200	AIx@75 (%)	Non-HIV		20.83	0.52	3.31	0.079
	PLWHIV		81.44	1.41				PLWHIV		24.14	1.76		
baPP (mmHg)	Non-HIV		45.77	0.42	1.18	0.432	Inflection Time (s)	Non-HIV		0.129	0.001	0.00	0.283
	PLWHIV		46.95	1.41				PLWHIV		0.125	0.003		
baMBPosc (mmHg)	Non-HIV		104.33	0.44	−1.46	0.359	Systolic Time (s)	Non-HIV		0.200	0.001	−0.008*	0.001
	PLWHIV		102.87	1.49				PLWHIV		0.192	0.002		
baMBPcalc (mmHg)	Non-HIV		98.63	0.43	−1.54	0.318	Tmax Backward (s)	Non-HIV		0.221	0.001	−0.006*	0.018
	PLWHIV		97.09	1.44				PLWHIV		0.215	0.002		
Heart Rate (beats/m)	Non-HIV		70.58	0.48	11.45	0.000	Tmax Forward (s)	Non-HIV		0.186	0.001	−0.010*	0.001
	PLWHIV		82.03	1.64				PLWHIV		0.176	0.003		
Stroke Volume (ml)	Non-HIV		73.88	0.53	−9.009*	0.000	Tstart Backward (s)	Non-HIV		0.055	0.001	−0.004*	0.039
	PLWHIV		64.88	1.81				PLWHIV		0.051	0.002		
Cardiac Output (l/min)	Non-HIV		5.12	0.03	0.11	0.356							
	PLWHIV		5.23	0.11									
Cardiac Index (l/min/m^2^)	Non-HIV		2.70	0.02	0.21	0.002							
	PLWHIV		2.90	0.06									
SVR (mmHg/l/min)	Non-HIV		1.25	0.01	−0.05	0.081							
	PLWHIV		1.20	0.03									
**Analysis of variables that depend on the calibration scheme**													
**Calibration scheme**													
		**baMBPosc/baDBP**				**baMBPcalc/baDBP**				**baSBP/baDBP**			
**Variables**	**Groups**	**MV**	**SE**	**Mean Difference**	***p*** **-value**	**MV**	**SE**	**Mean Difference**	***p*** **-value**	**MV**	**SE**	**Mean Difference**	***P*** **-value**
aoSBP (mmHg)	Non-HIV	135.2	0.64	−12.44	0.000	121.1	0.56	−6.36	0.002	120.3	0.53	−1.75	0.366
	PLWHIV	122.8	2.15			114.7	1.88			118.5	1.80		
aoDBP (mmHg)	Non-HIV	85.0	0.42	−2.53	0.096	84.5	0.42	−2.16	0.152	84.5	0.42	−1.58	0.296
	PLWHIV	82.4	1.42			82.3	1.41			82.9	1.42		
aoPP (mmHg)	Non-HIV	50.2	0.55	−9.87	0.000	36.6	0.44	−4.12	0.010	35.7	0.37	−0.15	0.912
	PLWHIV	40.4	1.87			32.5	1.50			35.6	1.25		
Inflection Pressure (mmHg)	Non-HIV	12.5	0.36	−3.82	0.003	9.2	0.28	−2.34	0.019	8.7	0.23	−0.86	0.308
	PLWHIV	8.7	1.21			6.8	0.93			7.8	0.79		
aoPb (mmHg)	Non-HIV	21.2	0.25	−4.87	0.000	15.3	0.20	−2.23	0.002	14.9	0.16	−0.09	0.877
	PLWHIV	16.3	0.86			13.0	0.68			14.9	0.56		
aoPf (mmHg)	Non-HIV	31.7	0.37	−6.31	0.000	23.3	0.30	−2.77	0.010	22.7	0.21	−0.31	0.686
	PLWHIV	25.4	1.25			20.5	1.01			22.4	0.73		
Pb/Pf (%)	Non-HIV	66.9	0.33	−2.75	0.022	65.7	0.34	−2.48	0.042	65.7	0.32	0.34	0.767
	PLWHIV	64.1	1.12			63.2	1.14			66.1	1.07		
aoPWV (m/s)	Non-HIV	8.3	0.03	−0.55	0.000	7.7	0.03	−0.31	0.002	7.7	0.02	−0.15	0.076
	PLWHIV	7.8	0.10			7.4	0.09			7.5	0.08		

*MV, men value; SE, standard error; PLWHIV, people living with HIV; baSBP, baDBP, baPP, brachial artery systolic, diastolic and pulse pressure; baMBPosc and baMBPcalc, brachial artery mean blood pressure directly derived by oscillometry and calculated, respectively; SVR, systemic vascular resistance; PWA, pulse wave analysis; tmax, time to maximal; Aix, augmentation index; aoSBP, aoDBP, aoPP, aortic systolic, diastolic and pulse pressure; Pb and Pf, aortic backward and forward pressure component; aoPWV, aortic pulse wave velocity; Mean Difference refers to, HIV- minus PLWHIV value; Covariates appearing in the modelswere evaluated as follows, age: 51.09 y; sex (1: females; 0: male): 0.49; BMI: 27.98 kg/m^2^; obesity (1: yes; 0: no): 1.02; current smoker (1: yes; 0: no): 0.17; CVD (1: yes; 0: no): 0.03; hypertension (1: yes; 0: no): 0.60; anti-hypertensive treatment (1: yes; 0: no): 0.52; dyslipidemia (1: yes; 0: no): 0.61; hyperlipidemia treatment (1: yes; 0: no): 0.26; diabetes (1: yes; 0: no): 0.09; anti-diabetic treatment (1: yes; 0: no): 0.07; hematocrit, 42.02%; glycemia, 98.82 mg/dl; creatinine, 0.8895 mg/dl; total cholesterol, 189.55 mg/dl; LDL-cholesterol, 113.90 mg/dl; HDL-cholesterol, 53.56 mg/dl; triglycerides, 139.84 mg/dl*.

#### Explanatory Capacity of HIV Infection of the Differences Between the Measured and Expected (Optimal) Value of Cardiovascular Variables

Cardiovascular variables obtained in PLWHIV and non-HIV subjects were expressed as relative difference (%) with respect to age-matched healthy subjects not exposed to CRFs (reference group). To build the reference group, we identified a healthy sub-population from the project database that included subjects who did not meet any of the following exclusion criteria: history of cardiovascular disease, use of baBP-, lipid- or glucose-lowering drugs, hypertension or high baBP levels during the non-invasive evaluation, smoking, diabetes, dyslipidemia, obesity (29–36, 41–44).

Once the reference group was built, age-related equations were obtained for mean values (Supplementary Table 5). To this end, we implemented parametric regression methods based on various types of mathematical models (e.g., fractional polynomials) (29–36, 41–44). This procedure provides different equations for each model to calculate age-related mean values, then, the most adjusted model was chosen to calculate individual's relative difference between the expected and the measure value: [(measured-expected)/expected]^*^100 (Supplementary Table 6).

Finally, multiple linear regression (stepwise) models were constructed considering (i) the relative differences between the measured and expected values for the cardiovascular (dependent) variables and (ii) the presence of HIV infection and CRFs (similar cofactors included in ANCOVA; independent variables) (Table 3, Supplementary Tables 7, 8).

Table 3Multiple linear regression models between (i) relative differences between measured and expected cardiovascular level [(%), dependent variable] and cardiovascular risk factors and HIV status (independent variables).

**Table:** 

**Analysis of variables that do not depend on the calibration scheme**											
**Brachial blood pressure, heart rate and PCA−derived parameters**						**Aortic wave analysis-derived parameterss**					
**Dep. V**	**Indep. V**	**Bu**	**Bs**	***p*** **-value**	**aR** ^**2**^	**Dep. V**	**Indep. V**	**Bu**	**Bs**	***P*** **-value**	**aR** ^**2**^
Heart Rate	Constant	−0.279		0.014	0.18	Systolic Time	Constant	0.120		0.002	0.06
	HIV+	0.166	0.259	0.000			HIV+	−0.046	−0.165	0.000	
	Age	−0.003	−0.199	0.000			Htc	−0.003	−0.120	0.002	
	Glyc	0.001	0.143	0.000			Smoking	−0.021	−0.092	0.017	
	Sex	0.088	0.228	0.000			CVD	0.043	0.084	0.025	
	Obesity	0.022	0.088	0.022		Tmax Backward	Constant	0.135		0.001	0.03
	HDL	−0.001	−0.118	0.004			Htc	−0.002	−0.102	0.008	
	Htc	0.005	0.090	0.024			CVD	0.048	0.099	0.010	
	TC	0.000	0.075	0.041			Age	−0.001	−0.106	0.007	
Stroke Volume	Constant	0.085		0.009	0.16		HIV+	−0.026	−0.099	0.011	
	Sex	−0.115	−0.307	0.000		Tmax Forward	Constant	0.144		0.002	0.05
	HIV+	−0.130	−0.208	0.000			HIV+	−0.059	−0.176	0.000	
	Age	0.002	0.157	0.000			Htc	−0.004	−0.123	0.001	
	Smoking	−0.045	−0.091	0.012			Smoking	−0.022	−0.083	0.032	
	Dyslip	−0.031	−0.080	0.049		AIx	Constant	0.585		0.086	0.08
Cardiac Index	Constant	0.144		0.003	0.19		Sex	0.210	0.178	0.000	
	BMI	−0.013	−0.364	0.000			TG	0.000	−0.089	0.017	
	Sex	0.065	0.182	0.000			HIV+	−0.207	−0.106	0.005	
	HIV+	0.072	0.122	0.001			Htc	−0.020	−0.118	0.005	
	Glyc	0.001	0.094	0.015		
	Anti−dyslip	−0.030	−0.074	0.045		
	HTN	0.076	0.212	0.003		
	Anti−HTN	−0.053	−0.149	0.033		
Systemic Vascular Resistance	Constant	0.273		0.001							
	HTN	0.046	0.132	0.001							
	Glyc	−0.001	−0.106	0.008							
	Htc	−0.004	−0.086	0.025							
	HIV+	−0.047	−0.082	0.037

**Table:** 

**Analysis of variables that depend on the calibration scheme**															
**Calibration scheme**															
	**baMBPcalc/baDBP**					**baMBPosc/baDBP**					**baSBP/baDBP**				
**Dep. V**	**Indep. V**	**Bu**	**Bs**	***p*** **-value**	**aR** ^**2**^	**Indep. V**	**Bu**	**Bs**	***p*** **-value**	**aR** ^**2**^	**Indep. V**	**Bu**	**Bs**	***p*** **-value**	**aR** ^**2**^
aoSBP	Constant	0.037		0.262	0.21	Constant	0.065		0.056	0.23	Constant	−0.005		0.902	0.23
	HTN	0.140	0.510	0.000		HTN	0.141	0.487	0.000		HTN	0.159	0.590	0.000	
	Sex	−0.054	−0.199	0.000		Sex	−0.053	−0.188	0.000		Anti−HTN	−0.081	−0.307	0.000	
	Anti−HTN	−0.077	−0.284	0.000		HIV+	−0.109	−0.232	0.000		BMI	0.005	0.172	0.000	
	HIV+	−0.061	−0.136	0.000		Anti−HTN	−0.080	−0.283	0.000		Age	−0.001	−0.151	0.000	
	BMI	0.003	0.103	0.005		BMI	0.002	0.080	0.027		Sex	−0.033	−0.123	0.000	
	————	————	————	————		————	————	————	————		Anti−dyslip	−0.034	−0.111	0.003	
	————	————	————	————		————	————	————	————		TC	0.000	0.070	0.046	
aoPP	Constant	−0.142		0.011	0.07	Constant	−0.049		0.353	0.10	Constant	−0.101		0.079	0.03
	Age	0.005	0.223	0.000		HIV+	−0.218	−0.220	0.000		BMI	0.005	0.092	0.021	
	HIV+	−0.115	−0.111	0.003		Age	0.004	0.180	0.000		Anti−dyslip	−0.051	−0.085	0.031	
	Sex	−0.058	−0.094	0.012		Sex	−0.054	−0.090	0.014		HTN	0.127	0.236	0.002	
	————	————	————	————		————	————	————	————		Anti−HTN	−0.092	−0.175	0.020	
Inflection Pressure	Constant	1.352		0.001	0.09	Constant	0.988		0.013	0.08	Constant	0.681		0.095	0.06
	Age	0.012	0.202	0.000		HIV+	−0.356	−0.143	0.000		Sex	0.215	0.154	0.000	
	Hyc	−0.038	−0.162	0.000		Htc	−0.029	−0.132	0.000		HIV+	−0.229	−0.099	0.009	
	HIV+	−0.334	−0.125	0.001		HDL	0.004	0.089	0.022		Htc	−0.021	−0.106	0.012	
	Glyc	−0.003	−0.094	0.013		Age	0.007	0.119	0.002		TG	0.000	−0.076	0.045	
	————	————	————	————		Glyc	−0.003	−0.101	0.009		————	————	————	————	
Pb	Constant	−0.143		0.020	0.08	Constant	0.101		0.203	0.11	Constant	−0.087		0.150	0.03
	Age	0.006	0.221	0.000		HIV+	−0.244	−0.225	0.000		BMI	0.004	0.075	0.060	
	HIV+	−0.131	−0.115	0.003		Age	0.005	0.198	0.000		Anti−dyslip	−0.054	−0.085	0.030	
	Sex	−0.063	−0.093	0.012		Sex	−0.061	−0.094	0.010		HTN	0.143	0.251	0.001	
	Smoking	−0.074	−0.081	0.031		Glyc	−0.002	−0.136	0.002		Anti−HTN	−0.111	−0.200	0.008	
	————	————	————	————		Diabetes	0.114	0.100	0.020		————	————	————	————	
Pf	Constant	−0.145		0.013	0.08	Constant	−0.057		0.294	0.11	Constant	−0.153		0.016	0.03
	Age	0.005	0.228	0.000		HIV+	−0.215	−0.207	0.000		HTN	0.121	0.240	0.002	
	Sex	−0.072	−0.110	0.003		Age	0.005	0.208	0.000		BMI	0.004	0.085	0.032	
	HIV+	−0.116	−0.106	0.005		Sex	−0.072	−0.115	0.002		Anti−HTN	−0.087	−0.175	0.020	
	Anti−DM	0.095	0.075	0.042							Age	0.001	0.082	0.046	
Pb/Pf	Constant	0.023		0.255	0.01	Constant	0.119		0.032	0.02	Constant	−0.009		0.679	0.03
	Smoking	−0.031	−0.095	0.014		HIV+	−0.057	−0.147	0.000		Sex	0.028	0.125	0.001	
	Glyc	0.000	−0.079	0.041		Dyslip	−0.023	−0.097	0.017		Age	−0.001	−0.125	0.001	
	————	————	————	————		Htc	−0.003	−0.083	0.031		Smoking	−0.026	−0.086	0.023	
aoPWV	Constant	0.031		0.139	0.18	Constant	0.125		0.000	0.18	Constant	0.017		0.440	0.23
	HTN	0.065	0.374	0.000		HIV+	−0.083	−0.260	0.000		BMI	0.003	0.188	0.000	
	Sex	−0.039	−0.226	0.000		Sex	−0.043	−0.222	0.000		HTN	0.083	0.525	0.000	
	HIV+	−0.044	−0.154	0.000		HTN	0.074	0.377	0.000		Age	−0.002	−0.264	0.000	
	Anti−HTN	−0.034	−0.200	0.004		Anti−HTN	−0.040	−0.211	0.002		Anti−HTN	−0.041	−0.266	0.000	
	BMI	0.002	0.103	0.005		Age	−0.001	−0.098	0.010		Sex	−0.021	−0.133	0.000	
	————	————	————	————		————	————	————	————		TC	0.000	0.079	0.023	

*Glyc, Glycaemia (1: yes, 0: no); Sex, 1 (female), 0 (male); Obesity, 1 (yes), 0 (no); TC, total cholesterol; HDL, high-density lipoprotein cholesterol; Htc, hematocrit; Smoking, current smoking (1: yes, 0: no); CVD, cardiovascular disease (1: yes, 0: no). Dyslip, dyslipidemia (1: yes, 0: no); BMI, body mass index; TG, triglycerides; HTN, hypertension (1: yes, 0: no); Anti-dyslip, Anti-dyslipidemia treatment (1: yes, 0: no); Anti-HTN, Anti-hypertension treatment (1: yes, 0: no). Anti-DM, anti-diabetic mellitus treatment (1: yes, 0: no); Dep. V, dependent variable; Indep. V, independent or explanatory variable. Bu and Bs, un- and standardized beta coefficient; aR^2^, adjusted R^2^ p value; Aix, aortic augmentation index; aoSBP and aoPP, aortic systolic and pulse pressure; Pb and Pf, backward and forward aortic component. Units of independent variables are the same than in Table 1. Only variables in which HIV infection (HIV+) was statistically significant as an explanatory variable are shown. A variance inflation factor <5 was selected to evaluate (discard) significant multicollinearity*.

Figure 1 highlights the relative difference between the measured and the expected value (ranked from highest to lowest for each calibration scheme) independently explained by the HIV infection.

#### Sample Size and Bootstrapping

A normal distribution was considered according to the central limit theorem, kurtosis, skewness coefficients distribution and the number of subjects studied (sample size >30) (45). The number of subjects included was higher than the minimum required sample size, both to build the reference group to obtain the mean values equations (included: 398, required size: 377) and to carry out association analyses (included: 1,086, required size: 103). The number of subjects studied was higher than the minimum number calculated for: α = 0.05, β = 0.20, anticipated effect size = 0.15 (medium) and a total number of predictors in the regression models = 7. Even in this adequate and conservative context (e.g., sufficient sample size, adjusted comparisons), when making comparisons and associations we performed Bootstrapping of the samples, as a strategy to evaluate whether potential differences and/or associations observed between cardiovascular variables and subject condition do maintain even after analyzing different random sampling settings (resampling with replacement from the original sample). In other words, with this mechanism, any initial p<0.05 may no longer be significant after the “fictional random re-sampling” (i.e., bootstrapping). This type of test obligates the investigators to consider only those significant *p* values that replicate in both statistical scenarios (the actual sample and bootstrapping sampling). To this end, Bootstrap-derived 95% confidence intervals (1,000 samples) were obtained applying bias-corrected and accelerated methods for computing lower and upper confidence interval limits. The difference between mean values or association was considered significant only if the bootstrapping-derived *p* value was < 0.05 or 95% confidence interval of regression coefficient, quantified by bootstrapping did not contain the 0 value. It should be taken into account that in all cases, the use of the Bootstrapping technique confirmed the results obtained before it was applied.

All analyses were performed using SPSS Software (v.26, IBM-SPSS Inc., IL, USA), MedCalc (v.14.8.1, MedCalc Inc., Ostend, Belgium) and NCSS 2020 (NCSS, Kaysville, UT). A *p* < 0.05 was considered statistically significant.

## Results

### Central Hemodynamics: Impact of Different Calibration Schemes

As expected, in all subgroups of subjects, the baMBPosc/baDBP calibration scheme resulted in higher levels of aoBP and waveform-derived parameters (Figure 1, Supplementary Table 1).

### PLWHIV and Central Hemodynamics: Impact of Different Calibration Schemes

Regardless of the exposure to CRFs, PLWHIV presented, compared to non-HIV subjects: (i) similar levels of cardiac output (increased heart rate but reduced stroke volume), (ii) similar baBP levels, (iii) a tendency to lower AIx levels, which dissipated after adjusting for heart rate (AIxHR75), and (iv) similar pressure inflection time (Table 2, Supplementary Tables 2–4).

PLWHIV presented, compared to HIV- subjects, a tendency of having lower levels of aoSBP, aoPP, inflection pressure, Pb, Pf, Pb/Pf ratio and aoPWV, which clearly reached significance (*p* < 0.001, *p* < 0.01, and *p* < 0.005) when using the baMBPosc/baDBP or baMBPcalc/baDBP as the calibration scheme (Table 2, Supplementary Tables 2–4). Conversely, aortic parameters calibrated by baSBP/baDBP did not reach statistical significance between the groups. Consequently, results were highly influenced by the calibration method and dictated whether PLWHIV is associated with lower aoBP levels and differences in wave-derived indexes with respect to HIV- subjects (Table 2, Supplementary Tables 2–4).

### HIV+ Status and Central Hemodynamics: Deviation From the Optimal Level in the Context of CRFs

Differences between the expected and measured values for all the baBP levels (baSBP, baDBP, baPP, baMBPcalc, baMBPosc) were not associated with the HIV infection (Table 3, Supplementary Tables 7–8). In contrast, differences between the measured and expected values of global hemodynamic parameters were positively (heart rate and cardiac index) and negatively (stroke volume and systemic vascular resistance) associated with the HIV infection, regardless of the exposure to CRFs. HIV infection was also negatively associated with systolic time, Tmax Backward and Forward and AIx, independently of CRFs (Table 3, Supplementary Tables 7–8).

When analyzing the explanatory capacity (β standardized coefficients) of HIV infection as independent variable in the context of other CRFs, “HIV+” variable becomes (i) the first position, when considering heart rate, Systolic Time and Tmax Forward (e.g., surpassing age and sex), (ii) second position, after sex, when considering stroke volume (e.g., exceeding age and smoking), and (iii) third position when analyzing cardiac index and AIx. In each scenario, HIV infection status exceeded the ability of other CRFs, such as hypertension or obesity, to explain the changes in the hemodynamic parameters (Table 3, Supplementary Tables 7, 8).

Table 3, Supplementary Tables 7, 8 present the regression models for those variables whose values depend on the calibration scheme. When baMBPosc/baDBP and baMBPcalc/baDBP schemes were used, HIV was negatively associated with relative differences in aoSBP, aoPP, Inflection Pressure, Pb, Pf and aoPWV. The presence of HIV was only associated with the relative differences in the Pb/Pf ratio when the baMBPcalc/baDBP was the calibration scheme. With the sole exception of “Inflection Pressure,” HIV was not associated with the differences in the variables derived from the aoBP waveform analysis when baBSP/baDBP was used. Consequently, calibration by the baSBP/baDBP method was unable to reveal any cardiovascular variation associated with HIV infection.

When analyzing the position compared to other explanatory variables (e.g., CRFs) of the central hemodynamic variations (β standardized coefficients), while HIV infection positioned in the first place for the following variables: aoPP, Inflection Pressure, Pb, Pf, Pb/Pf and aoPWV (e.g., exceeding age and smoking) when considering baMBPosc/baDBP as the calibration method, it positioned in the third place when considering aoSBP. In each mentioned model, HIV+ status exceeded the relative weight of other CRFs, such as hypertension and obesity (Table 3, Supplementary Table 8).

When considering baMBPcalc/baDBP calibration scheme, the presence of HIV infection continued to surpass important CRFs in the explanatory ability, but it was no longer the main explanatory variable for any of the dependent variables analyzed (Table 3, Supplementary Table 8).

Finally, baSBP/baDBP calibration method only retains the presence of HIV infection as an explanatory variable of the Inflection Pressure behind other variables (Table 3, Supplementary Table 8). Interestingly, although HIV infection was not the main explanatory variable for aoSBP levels (regardless of the calibration scheme used), it was only exceeded by the presence of arterial hypertension, sex, or anti-hypertensive treatment, and in every case, its explanatory ability exceeded important CRFs (e.g., obesity, diabetes, dyslipidemia, smoking) (Table 3, Supplementary Table 8).

### HIV+ Status and Central Hemodynamics: Comparative Analysis of Calibration Schemes

Figure 1 shows the percentage of variation between the measured and optimal value of the cardiovascular variables for each of the calibration schemes that could be explained by the HIV status (values ranked from the highest to the lowest value). Additionally, Figure 1 presents comparatively the differences depending on the calibration scheme used. Regardless of other CRFs the presence of HIV infection explained up to 35.6% of the differences between the measured and the expected value of the cardiovascular variables.

In general, when baMBPosc/baDBP was the calibration scheme, the HIV infection was able to explain a greater percentage of the differences of the cardiovascular variables compared to other calibration methods, regardless of the variable analyzed (Figure 1). However, deviations from the expected value of certain variables were explained by HIV infection for (i) Inflection Pressure regardless of the calibration method, (ii) Pb, Pf, aoPP, aoSBP, aoPWV when calibrating by baMBPosc/baDBP or baMBPcalc/baDBP, and (iii) Pb/Pf when baMBPosc/baDBP was the selected calibration scheme (Figure 1).

Table 3, Figure 1 show a hierarchical order between the calibration schemes, since the differences explained by HIV status begin to decrease in magnitude until they are no longer significant when calibrating sequentially by baMBPosc/baDBP, baMBPcalc/baDBP and baSBP/baDBP.

## Discussion

The Main Findings of Our Study can be Summarized as Follows:

First, despite presenting similar levels of baBP than CRFs-matched non-HIV subjects, PLWHIV presented significantly lower levels of aoSBP and aoPP. The lower levels of aoSBP and aoPP would be determined by lower magnitudes of Pf and Pb, as well as by lower reflection magnitude (Pb/Pf) and aoPWV. These results were specifically noted when using the calibration approaches recommended in the current literature (baMBP/baDBP) (16) and were not observed when calibrating by the baSBP/baDBP approach. As was hypothesized, the peripheral signal calibration scheme is a determining factor when assessing central hemodynamic variables in PLWHIV.

Second, HIV infection was an important explanatory factor of the differences of the levels of central hemodynamic variables, with respect to the expected value in healthy subjects not exposed to CRFs, exceeding in relative importance to classical CRFs (e.g., obesity, diabetes, dyslipidemia, smoking). Moreover, the presence of HIV infection was the main explanatory variable for variations in central hemodynamics when using the baMBPosc/baDBP calibration (followed by the baMBPcalc/baDBP scheme), exceeding CRFs such as age, sex, and hypertension. Consequently, the HIV status would be able to better explain variations in cardiovascular characteristics than classic CRFs. However, the relative importance of HIV status as an explanatory variable is highly dependent on the calibration scheme used, at the time that its explanatory capacity is reduced or even lost when calibrating by baSBP/baMBP. Therefore, the calibration scheme not only affects the absolute differences in the aortic cardiovascular variables measured in PLWHIV vs. non-HIV subjects, but also the relationship between these variables and the different (potential) explanatory variables of their values.

Third, within the central hemodynamic variables, HIV infection is more associated with variations in waveform-derived indexes (e.g., inflection pressure, AIx) than aoBP levels. In addition, regardless of the cardiovascular variable analyzed, baMBPosc/baDBP calibration determined that the presence of HIV infection explains a greater percentage of the differences of the cardiovascular variables.

These observations stressed out the relevance of reaching a consensus and systematization on the methodology used for the non-invasive assessment of central hemodynamics, since otherwise “different results” can be obtained despite analyzing the same patient. Consequently, it is not surprising, that controversial results have been reported on central hemodynamics between subjects with and without HIV infection (9–15). However, some considerations need to be pointed out. We found that regardless of exposure to CRFs, PLWHIV showed a tendency to present lower levels of stroke volume and cardiac index, higher heart rate, all of which led them to present unchanged cardiac output. The high heart rate observed in PLWHIV is consistent with previous reports (9, 13, 14). Ngatchou et al. (13) and Vlachopoulos et al. (14) reported that heart rate was higher in PLWHIV compared to non-HIV subjects, by an absolute mean value of 10 and 6.4 beats/min, respectively, results that are in line with our study in where the heart rate of PLWHIV was 11 beats/min more in average than the heart rate of HIV- subjects (Table 2).

Although it might be unexpected that HIV infection is associated with a “better hemodynamic profile,” lower levels of aoBP and wave reflection indexes have already been reported in previous studies (9, 13, 14). Ngatchou et al. (13) in Cameroon, measured waveform-derived parameters (e.g., AIx) in apparently healthy subjects (*n* = 96, 41 ± 12 years) and untreated PLWHIV (*n* = 108, 39 ± 10 years). Authors reported that age- and sex-adjusted AIx was significantly lower in PLWHIV compared to non-HIV subjects (6 ± 4 vs. 8 ± 7%, *p* = 0.01). Vlachopoulos et al. (14) in Greece studied PLWHIV (*n* = 51) with a recent HIV infection, free of antiretroviral treatment, and non-HIV subjects (*n* = 35), matched for age, sex, and smoking. The authors reported that while aortic stiffness was similar in the two groups (*p* = 0.74), aoSBP (by 4.6 mmHg, *p* = 0.059), aoDBP (by 5.7 mmHg, *p* = 0.017), Tr (the time the pulse wave needs to travel to the periphery and return to meet the incident wave), AIx (by 6.4%, *p* = 0.048) and augmentation pressure (by 3.3 mmHg, *p* = 0.010) were lower in PLWHIV. Consequently, these authors provided further evidence of PLWHIV having reduced aoBP and wave reflections, but similar aortic stiffness, at least in the early stages of the disease. Importantly, these authors calibrated the pressure waveform using the baSBP/baDBP scheme, so it would be expected that potential aoBP differences in PLWHIV vs. non-HIV subjects could have been greater (in the early stages) and/or notoriously significant if another calibration scheme were used (14).

Taken together, reduced aoBP levels in the setting of similar baBP in PLWHIV would suggest that both HIV infection and/or highly active antiretroviral therapy play a role in hemodynamics, with differential effects on different locations of the vasculature (central vs. peripheral vessels). As was discussed by Martínez-Ayala et al. (12) lower aoBP levels may be caused by a peripheral vasodilation of small and medium-sized arteries, possibly induced by prostaglandins and other inflammatory cytokines associated with the chronic HIV infection. The vasodilation effect on peripheral reflection sites (e.g., arterial bifurcations) might cause a reduced Pb and Pb/Pf, and a reduced contribution to aoSBP.

In previous works performed on healthy subjects of the general population we showed that peripheral waveform calibration with the “baMBPosc/baDBP” scheme resulted in higher aoBP values (27–29). In this work we confirm these results. In fact, in all subgroups, the baMBPosc/baDBP calibration scheme resulted in higher levels of aoBP and waveform-derived indexes (Figure 1, Supplementary Table 1).

### Clinical Importance

By using an automated oscillometric device and performing rigorous analyses of different groups, our study revealed that despite similar levels of brachial pressure, PLWHIV presented lower aoBP levels, explained by lower wave reflections and arterial stiffness. Additionally, compared to other CRFs, HIV infection demonstrated the highest explanatory capacity for variations in central hemodynamics.

Taking into account that the baMBP/baDBP method is currently the most recommended calibration scheme, PLWHIV would have a condition of equal or even lower ventricular afterload compared to non-HIV subjects.

Additionally, these results evidenced that the calibration approach is an important determinant of the results non-invasively obtained in central hemodynamics in PLWHIV. Our study strongly emphasizes the need for methodological transparency and consensus for the non-invasive assessment of central hemodynamic parameters in PLWHIV, and possibly in the general population.

### Strengths and Limitations

First, our non-invasive approach is unable to identify the “ideal” calibration strategy, since it requires invasive (catheterism) vs. non-invasive agreement analysis. By performing agreement analyses, we would be able to reveal which calibration method is the one that achieves a more accurate and reliable quantification of the blood pressure values existing in the aortic root. However, invasive aoBP measurements are commonly not performed in subjects for obvious ethical reasons (when not performed due to other strict clinical indications). Second, our results are derived from cross-sectional studies, and therefore our observations do not allow us to know which calibration method has the greatest predictive ability of future cardiovascular events and/or disease. Additionally, it provides no data on longitudinal HIV-related temporal variations in variables of interest. Third, in contrast to other studies (9), we have not divided the subjects according to whether they were or were not under pharmacologic treatment, or to the time of being exposed to it. However, previous studies showed that HIV treatment does not contribute significantly to changes in the aoBP associated with HIV infection (10). Fourth, we have not considered the HIV duration as possible determinant variable of the central hemodynamic parameters, given that different studies showed that there were no significant differences in baBP, aoBP, aoPWV or AIx between patients with shorter vs. longer duration of HIV infection (13).

Finally, our population of PLWHIV can be considered characteristic of a population of subjects in outpatient care. Our cohort has an acceptable rate of antiretroviral treatment (46, 47). Moreover, in our cohort, the coinfection rate with cytomegalovirus (48) and hepatitis B (49) was lower than that in other cohorts, possibly because of differences in HIV transmission routes, age, or geographical differences.

## Conclusions

In PLWHIV and non-HIV subjects, the baMBPosc/baDBP calibration scheme resulted in higher levels of aoBP and waveform-derived parameters. Despite similar levels of baBP, PLWHIV presented lower levels of aoSBP and aoPP compared to non-HIV subjects. Lower aoSBP and aoPP would be determined by both lower Pf and Pb, as well as by the lower levels of Pb/Pf and aoPWV. These results were only observed when using the calibration approach currently recommended (baMBP/baDBP), while these differences were not revealed by the baSBP/baDBP calibration scheme. PLWHIV showed a tendency to present lower levels of stroke volume and cardiac index, higher heart rate, and unchanged cardiac output.

The presence of HIV infection was shown to be an important determinant of the differences in the levels of the central hemodynamic variables, with respect to the expected value in healthy subjects not exposed to CRFs, exceeding important classical CRFs. The presence of HIV infection was the main explanatory variable for variations in central hemodynamics when using the baMBPosc/baDBP calibration, followed by the baMBPcalc/baDBP approach.

The calibration approach is an important determinant of the results obtained in central hemodynamics in PLWHIV. Our study strongly emphasizes the need for transparency and consensus in the methodologies employed for the non-invasive assessment of aoBP levels and waveform-derived indexes in PLWHIV and possibly in the general population.

## Data Availability Statement

The original contributions presented in the study are included in the article/Supplementary Material, further inquiries can be directed to the corresponding authors.

## Ethics Statement

The studies involving human participants were reviewed and approved by Comité de Ética de Investigación. Hospital Dr. Héctor M. Cura, Olavarría, Provincia de Buenos Aires, Argentina. The patients/participants provided their written informed consent to participate in this study.

## Author Contributions

AD, MG, YZ, and DB contributed to conception and design of the study. AD, FS, and MG performed the cardiovascular non-invasive recordings and constructed, and organized the database. YZ and DB performed the statistical analysis. AD, MG, and DB wrote the first draft of the manuscript. AD, MG, JT, YZ, FS, and DB performed revisions and critically discussed the complete manuscript. All authors, read, and approved the submitted version.

## Funding

This research was partially funded by extra-budgetary funds provided by the CUiiDARTE Center (YZ and DB).

## Conflict of Interest

The authors declare that the research was conducted in the absence of any commercial or financial relationships that could be construed as a potential conflict of interest.

## Publisher's Note

All claims expressed in this article are solely those of the authors and do not necessarily represent those of their affiliated organizations, or those of the publisher, the editors and the reviewers. Any product that may be evaluated in this article, or claim that may be made by its manufacturer, is not guaranteed or endorsed by the publisher.

## Glossary

Aix: Aortic augmentation index
AIxHR75: Heart rate-adjusted aortic augmentation index (for heart rate equal 75 beats/min)
aoBP: Aortic blood pressure
aoPWV: Aortic pulse wave velocity
aoSBP: Central aortic systolic blood pressure
aoPP: Central aortic pulse pressure
aoDBP: Central aortic diastolic blood pressure
baBP: Brachial artery blood pressure
baDBP: Brachial artery diastolic blood pressure
baMBP: Brachial artery mean blood pressure
baMBPcalc: Brachial artery mean blood pressure calculated using equations
baMBPcalc/baDBP: Calibration to baDBP and calculated brachial mean blood pressure
baMBPosc: Brachial artery mean blood pressure measured directly by oscillometry
baMBPosc/baDBP: Calibration to baDBP and oscillometry-derived brachial mean blood pressure
baPP: Brachial artery pulse pressure
baSBP: Brachial artery systolic blood pressure
baSBP/baDBP: Calibration to brachial systolic and diastolic pressure (systolic-diastolic calibration)
CRFs: Cardiovascular risk factors
HIV: Human immunodeficiency virus
HIV-: Non-HIV subjects or non-HIV infection
HIV+: Subjects with HIV infection
Pb: Peak or maximal amplitude of backward (reflected) wave component
Pf: Peak or maximal amplitude of forward (incident) wave component
PLWHIV: People living with human immunodeficiency virus
Tmax Backward: Time from the “foot wave” to the peak of backward (reflected) wave component
Tmax Forward: Time from the “foot wave” to the peak of forward (incident) wave component
Tstart Backward: Time from the “foot wave” to the initial phase (arrival) of the backward component.
